# Photoacoustic effects in nanocomposite structure ‘porous silicon-liquid’

**DOI:** 10.1186/1556-276X-7-411

**Published:** 2012-07-23

**Authors:** Dmitriy Andrusenko, Mykola Isaiev, Andrey Kuzmich, Vladimir Lysenko, Roman Burbelo

**Affiliations:** 1 Faculty of Physics, Taras Shevchenko National University of Kyiv, 64, Volodymyrs'ka St., Kyiv, 01601, Ukraine; 2 Institut des Nanotechnologies de Lyon (INL), UMR-5270, CNRS, INSA de Lyon, Universitй de Lyon, bat. Blaise Pascal, 7 Av. Jean Capelle, Villeurbanne, 69621, France

**Keywords:** Porous silicon, Photoacoustic and photothermal phenomena, Nanocomposite structures

## Abstract

Photoacoustic effect in nanocomposite structure ‘porous silicon-liquid’ has been investigated. Main mechanisms involved in the formation of photoacoustic signal in such structures have been experimentally studied. Liquids with different viscosity (ethanol and acetone) filling the nanopores have been used. A proposed mathematical model describing the photoacoustic signal formation was found to be in good agreement with the experimental results. The role of thermally induced pressures provoked by the liquids confined inside the nanopores in the photoacoustic process has been analyzed.

## Background

‘Porous matrix-liquid’ structures are widely used in such fields of science and technology as medicine, biology, geology, chemistry, etc. [1]. This is important to know mechanical, thermal, optical, and other properties of these structures from an application point of view. Investigations of such properties allow to obtain a lot of useful information about the hosting porous matrix, behavior of the liquids in the confining pore spaces, (for instance, properties of nanofluids), as well as about the whole composite porous matrix-liquid structure.

Photoacoustic (PA) techniques have proven to be capable for studying of porous materials [2] and layered structures [3]. This is mainly due to the fact that, in most cases of their practical implementations, they are nondestructive. In particular, it has been already demonstrated the possibility of using PA techniques for the study of fluid migration in porous materials [4]. Moreover, the PA signal detected by a piezoelectric registration was shown [5] to be sensitive both to thermally induced pressures (TIP) of the liquids localized inside the pores of the composite ‘porous silicon-liquid’ system and to the pressure relaxation phenomena. Thus, understanding of the PA transformation effects in the porous materials with empty or filled pores enables development of new experimental and diagnostic methods (including *in situ* methods in the manufacturing process) for characterization of such materials and composites.

In this paper, photoacoustic signals recorded with a piezoelectric transducer in the nanocomposite structure porous silicon-liquid are theoretically simulated and experimentally measured. In particular, the influence of thermally induced pressures of the liquid in the pores on shape and parameters of the photoacoustic response is analyzed in detail.

## Methods

**Figure 1:**
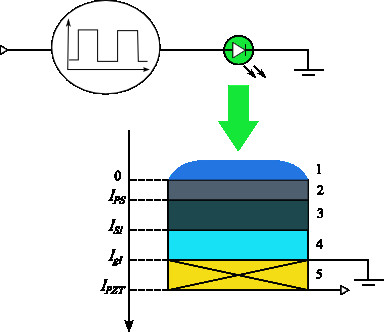
**Scheme of the studied structure.** (1) A liquid drop on the sample surface; (2) porous silicon layer; (3) bulk silicon substrate; (4) buffer glass-ceramic layer; and (5) piezoelectric transducer

A sandwich layered structure shown in Figure 1 is investigated. Mesoporous silicon surface covered with a layer of transparent liquid (ethanol, acetone) was illuminated by a blue LED (the wavelength corresponding to the maximum spectral energy density was 470 nm). Rectangular modulation of the exciting light intensity has been performed with a frequency of 78.2 Hz and duty cycle of 0.5. Thermoelastic stresses occurring in the structure have been deduced from the recorded time-dependent voltage of the piezoelectric transducer (analogue for PZT-5A, thickness 200 μm).

The mesoporous silicon layers were prepared by anodic etching. Highly boron-doped p^+^-type (0.01 to 0.02 Ω.cm) double-sided polished (100)-oriented Si wafers were electrochemically treated. Etching solution contained a 1:1 volume mixture of concentrated aqueous HF acid (49%) and pure ethanol. A constant anodic current density depending on the desirable porosity value was applied for various times to ensure different thicknesses of the final porous layer. A permanent stirring of the etching solution was applied in order to evacate hydrogen bubbles formed during the anodization process.

### Modeling

#### Temperature distribution

Absorption depth of electromagnetic radiation (with the wavelength below 600 nm) in the mesoporous silicon is about a few microns. Therefore, the case of near-surface absorption has been considered. The *Z* axis is directed in the depth of the sample, and the coordinate origin is situated on the top surface of the porous silicon layer. The temperature distribution inside the sample can be described by heat diffusion equation:

(1) $$c\rho \frac{\partial T}{\partial t}=\frac{\partial }{\partial z}\left(\chi \frac{\partial T}{\partial z}\right)\text{,}$$

where *c* and *ρ* are the heat capacity and volume density, respectively; *χ*, the thermal conductivity. The boundary conditions for this equation are as follows:

${T|}_{z=-\infty }\to 0$, where the fluid layer is thermally thick, and thermal perturbation does not penetrate to the top boundary of the liquid;

${\left(\partial T/\partial z\right)|}_{z=l_{\text{Si}}}=0$, where thermal conductivity of the glass ceramics is much smaller than the thermal conductivity of bulk silicon, so we assumed that the heat did not penetrate in it;

${\chi \left(\partial T/\partial z\right)|}_{z=0+0}-{\chi \left(\partial T/\partial z\right)|}_{z=0-0}=I\cdot f\left(t\right)$, where electromagnetic irradiation is absorbed at the top surface of the porous silicon and where *I* is the irradiation intensity; *f*(*t*) is the function describing temporal dependence (modulation) of the irradiation intensity.

 Periodic rectangular modulated irradiation with cycle duty of 0.5 was used in our experiment:

(2) $$f\left(t\right)=\{\begin{array}{cc} 1 & 0<t\leq T_{p}/2\\ 0 & T_{p}/2<t\leq T_{p} \end{array},\;f\left(t\right)=f\left(t+T_{p}\right)$$

According to this,

(3) $$f\left(t\right)=\sum_{n=-\infty }^{+\infty }f_{n}\cdot e^{i\omega_{n}t},f_{n}=\frac{1}{T_{p}}\int_{0}^{T_{p}}f\left(t\right)e^{-i\omega_{n}t}dt\text{,}$$

where *ω*_*n*_ =2π*n*/*T*_*p*_ (*n* is the integer number); *n* = 0 in the sum term corresponding to the averaged intensity distribution for one period has been omitted.

Thus, the depth and time-dependent temperature in the sample can be written in the following form:

(4) $$T\left(z,t\right)=\sum_{n=-\infty }^{+\infty }T_{n}\left(z\right)\cdot e^{i\omega_{n}t}\text{.}$$

The equations for temperature harmonic components can be obtained by substitution of expressions (Equations 2 and 3) in Equation 1 with the following boundary conditions:

(5) $$\begin{array}{c} \frac{\partial }{\partial z}\left(\chi \frac{\partial T_{n}}{\partial z}\right)-c\rho i\omega_{n}T_{n}=0\\ {T_{n}|}_{z=-\infty }\to 0\\ {\left(\partial T_{n}/\partial z\right)|}_{z=l_{\text{Si}}}=0\\ {\chi \left(\partial T_{n}/\partial z\right)|}_{z=0+0}-{\chi \left(\partial T_{n}/\partial z\right)|}_{z=0-0}=I\cdot f_{n} \end{array}$$

Solution of these equations for the simulated layer structure shown in Figure 1 can be represented as follows:

(6) $$T_{n}(z)=\left\{ \begin{array}{cc} A_{n}^{l}e^{\mu_{n}^{l}z} & z<0\\ A_{n}^{\text{PS}}e^{\mu_{n}^{\text{PS}}z}+B_{n}^{\text{PS}}e^{-\mu_{n}^{\text{PS}}z} & 0<z<l_{\text{PS}}\\ A_{n}^{\text{Si}}e^{\mu_{n}^{\text{Si}}z}+B_{n}^{\text{Si}}e^{-\mu_{n}^{\text{Si}}z} & l_{\text{PS}}<z<l_{\text{Si}} \end{array}\text{,}\right.$$

where $\mu_{n}^{j}={\left(i\omega_{n}c_{j}\rho_{j},/,\chi_{j}\right)}^{1/2}\text{.}$

The constants $A_{n}^{l}$, $A_{n}^{\text{PS}}$, $B_{n}^{\text{PS}}$, $A_{n}^{\text{Si}}$ and $B_{n}^{\text{Si}}$ can be obtained from the boundary conditions.

#### Thermally induced pressure

At quasi-stationary approximation, the thermally induced pressure (TIP) distribution for a liquid infiltrated in the mesoporous layer can be described by filtration equation [6]. Considering thermal expansion of the liquid, this equation can be written as follows:

(7) $$\frac{\partial p}{\partial t}-\frac{K}{\eta \beta \varepsilon }\frac{\partial^{2}p}{\partial z^{2}}=\frac{\beta_{\mathrm{Tl}}}{\beta }\frac{\partial T}{\partial t}\text{,}$$

where *ε* is the porosity, *β*_*Tl*_ is the coefficient of volume thermal expansion of the liquid, *K* is the fluid permeability of the porous silicon, *η* is the liquid’s viscosity, *β* is the liquid’s compressibility.

Boundary conditions for Equation 6 are the following:

(8) $${p|}_{z=0}=0{\frac{\partial p}{\partial z}|}_{z=l_{\text{por}}}=0\text{.}$$

Taking into account linear approximation for Equation 3, one can obtain expression for the liquid pressure inside the pores:

(9) $$p\left(z,t\right)=\sum_{n=-\infty }^{+\infty }p_{n}\left(z\right)e^{i\omega_{n}t}$$

The equation for pressure harmonic component can be obtained by substitution of expressions (Equations 3 and 8) in Equations 6 and 7:

(10) $$\begin{array}{c} \text{i}\omega_{n}p_{n}-\frac{K}{\eta \beta \varepsilon }\frac{\partial^{2}p_{n}}{\partial z^{2}}=\frac{\beta_{\text{Tl}}}{\beta }\text{i}\omega_{n}T_{n}\\ {p_{n}|}_{z=0}=0{\frac{\partial p_{n}}{\partial z}|}_{z=l_{\text{por}}}=0 \end{array}$$

Solution of this equation can be represented as follows:

(11) $$p_{n}=A_{n}^{1}e^{-\gamma_{n}z}+A_{n}^{2}{\text{e}}^{+\gamma_{n}z}+\text{i}\omega \frac{3\beta_{T_{l}}\eta }{\kappa \left(\gamma_{n}^{2}-\mu_{\text{por}}^{2}\right)}T_{n}\text{,}$$

where

(12) $$\gamma_{n}^{2}=i\omega_{n}\frac{\eta \beta \Pi }{\kappa }\text{.}$$

The constants $A_{n}^{1}$ and $A_{n}^{2}$ can be obtained from the boundary conditions.

#### Photoacoustic signal formation

Elastic deformations in the nanocomposite structure porous silicon-liquid can appear under its heating by non-stationary irradiation as a result of a thermoelastic mechanism. According to the equation [7] for quasi-stationary approximation, the source of thermoelastic force in such structures can be presented as follows:

(13) $$\sigma_{T}\left(z,t\right)=\frac{\alpha_{T}\left(z\right)E\left(z\right)}{1-\nu \left(z\right)}T\left(z,t\right)+\varepsilon p\left(z,t\right)\text{.}$$

This source consists of two components: (1) the thermoelastic stresses of the porous matrix and monocrystalline Si wafer (first term) as well as (2) the TIPs of the liquid inside the pores (second term).

Thus, the elastic stresses in the whole sample can be represented as follows:

(14) $$\sigma \left(z,t\right)=\int_{0}^{l_{\text{Si}}}\sigma_{T}\left(z^{\prime },t\right)G\left(z,z^{\prime }\right)dz^{\prime }\text{,}$$

where *G*(*z*, *z′* ) is the elasticity Green's function depending on elasticity parameters and geometry of the investigated sample. This function can be easily obtained for the case of quasi-stationary approximation (see, for example, reference [7]) using reciprocity theorem and Kirchhoff-Love theory:

(15) $$G\left(z,z^{\prime }\right)=\frac{E\left(z\right)}{1-\nu \left(z\right)}\left(\frac{a_{1}z^{\prime }-a_{2}+\left(a_{1}-a_{0}z^{\prime }\right)z}{a_{0}a_{2}-a_{1}^{2}}\right)\text{,}$$

where $a_{i}=\int_{0}^{l}\frac{E\left(z\right)}{1-\nu \left(z\right)}z^{i}dz\text{.}$

If the polarization axis is perpendicular to the PZT surface, then time evolution of the photoacoustic signal shape at the PZT electrodes can be presented in this form:

(16) $$\begin{array}{l} \Delta \phi \left(t\right)∼\int_{l_{\text{el}}}^{l_{\text{PZT}}}\sigma \left(z,t\right)dz=\int_{l_{\text{el}}}^{l_{\text{PZT}}}\int_{0}^{l_{\text{Si}}}\sigma_{T}\left(z^{\prime },t\right)G\left(z,z^{\prime }\right)dz^{\prime }dz\\ =\int_{0}^{l_{\text{Si}}}\sigma_{T}\left(z^{\prime },t\right)g\left(z^{\prime }\right)dz^{\prime } \end{array}\text{.}$$

where $g\left(z^{\prime }\right)=\int_{l_{\text{el}}}^{l_{\text{PZT}}}G\left(z,z^{\prime }\right)dz$is the function describing the voltage excitation performance depending on a point at which the elastic force is applied.

## Results and discussion

**Figure 2:**
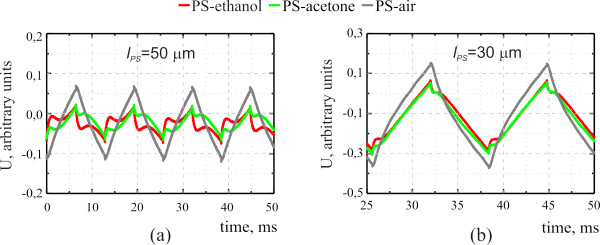
**Time-dependent PZT voltage measured for composite porous silicon-liquid structure for liquids with different viscosities.** Porosity of the used porous Si layer is 65% and its thicknesses are (**a**) 50 and (**b**) 30 μm

**Figure 3:**
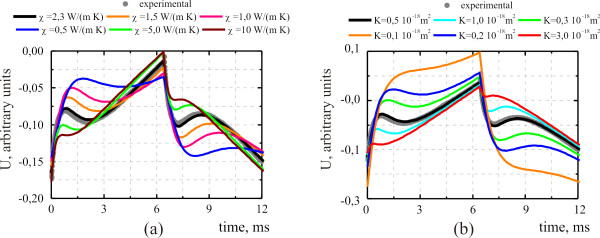
**Theoretical time-resolved photoacoustic signal.** (**a**) Influence of liquid viscosity and (**b**) influence of porous matrix permeability to liquids

Figure 2 shows typical experimental curves corresponding to the voltage oscillations of the used piezoelectric transducer (PA signal) recorded on the nanocomposite structure porous silicon-liquid for two different thicknesses of the porous layer. Liquids with different viscosity values (alcohol and acetone) were used to fill the nanopores of the porous matrix. The experimental PA signals obtained for the mesoporous silicon layer with empty pores (‘porous silicon-air’ structure) are shown for comparison. Simulated PA signals for the nanocomposite structure porous silicon-liquid are presented in Figure 3 for different values of thermal conductivity and fluid permeability of the porous matrix and compared with the corresponding experimental curves.

As one can see from Figures 2 and 3, the PA signal obtained for the porous Si-liquid structure is significantly different from the PA signal obtained on the similar structure without liquid (see reference [8], for example). In particular, at least two main characteristic features of the transient PA signals recorded from the nanocomposite structure porous Si-liquid can be noted: (1) a sharp initial rise is followed by (2) a more or less pronounced bend. These features become to be well resolved especially for relatively high porosity and larger thickness values, and the following characteristic sequence can be observed: sharp rise -maximum -bend -minimum -additional slight signal growth. These features are ensured by the TIP appearance (first feature) and relaxation (second feature) of the liquid inside the pores. The TIP arises due to significant differences between the parameters of the liquid and solid matrices (in particular, thermal expansion coefficients and compressibility). The time positions of these features depend on the thermophysical properties of liquid and solid matrix composites, fluid viscosity, the permeability *K*, and the thickness of the porous layer.

As we can see in Figure 3a, the first feature shifts toward the lower time range when thermal conductivity increases. It can be explained by the fact that thermal energy achieves more rapidly in Si wafer. Consequently, it leads to (a) the decrease of the liquid heating and (b) the general changes of relation between thermoelastic stresses and TIP contributions in the resulting structural deformations. Figure 3b illustrates influence of porous matrix permeability on the temporal shape of the PA signal. The first feature is shifted to the lower time edge with increasing permeability. The similar qualitative result is observed if the liquid viscosity decreases. This is due to the fact that the TIP relaxation time increases as a result of decreasing dissipative forces. Taking into account all these effects, the experimental PA signals can be perfectly fitted by the mathematical model described previously and by considering general thermophysical properties of the porous matrix-liquid composite, viscosity, permeability *K*, and thickness of the porous layer (for example, see comparison between experimental and simulated signals shown in Figure 3).

**Figure 4:**
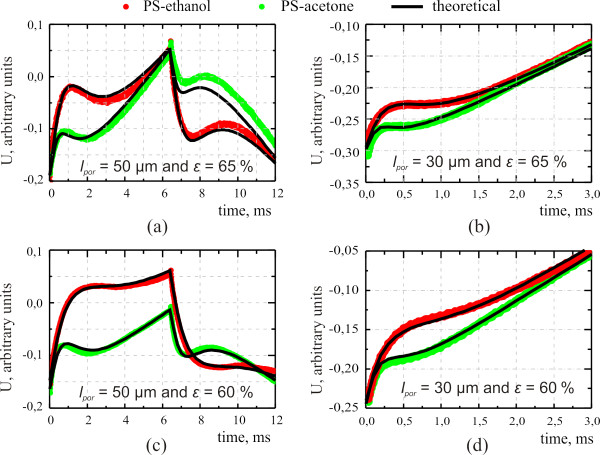
**Time-dependent voltage of the piezoelectric transducer.** Structural parameters of the porous silicon layers are (**a**) *l*_*por*_ = 50 μm and *ε* = 65%; (**b**) *l*_*por*_ = 30 μm and *ε* = 65%; (**c**) *l*_*por*_ = 50 μm and porosity *ε* = 60%; and (**d**) *l*_*por*_ = 30 μm and *ε* = 60%

Several fitting results of the time-resolved PA signals are shown in Figure 4. The order of magnitude of the porous Si permeability for well wetting liquids is found to be about 10^−18^ m^2^ which is in good agreement with the value determined previously from gas permeability measurements [9]. Thermal conductivity values of our nanocomposite structure (approximately 1 to 3 W/(m K) for different samples) correspond quite well to the experimental values of thermal conductivity of porous silicon reported earlier [10].

## Conclusions

In this paper, experimental and theoretical investigations of photoacoustic effect in nanocomposite porous silicon-liquid structures have been reported. The role of thermal-induced pressures of liquids confined inside the nanopores in formation of the photoacoustic signal has been highlighted. The influence of liquid and porous matrix parameters on the temporal shape of the photoacoustic signal has been demonstrated.

## Competing interests

The authors declare that they have no competing interests

## Authors’ contributions

DA made an experimental setup and experimental measurements. MI made the mathematical model and made modeling for the experimental conditions. AK made a modification of experimental setup and took part in fitting of experimental results. VL participated in the samples' fabrication and in general data analysis. RB coordinated the project and data analysis. All authors took part in experimental and modeling data analysis and discussions. All authors read and approved the final manuscript.
